# ﻿A taxonomic synopsis of Heliotropiaceae and new combinations in *Heliotropium* from Thailand

**DOI:** 10.3897/phytokeys.232.103647

**Published:** 2023-09-21

**Authors:** Kanokorn Rueangsawang, Pranom Chantaranothai

**Affiliations:** 1 Department of Biology, Faculty of Science, Ramkhamhaeng University, Bangkok 10240, Thailand Ramkhamhaeng University Bangkok Thailand; 2 Applied Taxonomic Research Center, Department of Biology, Faculty of Science, Khon Kaen University, Khon Kaen 40002, Thailand Khon Kaen University Khon Kaen Thailand; 3 Royal Botanic Gardens, Kew, Richmond, Surrey TW9 3AE, UK Royal Botanic Gardens Richmond United Kingdom

**Keywords:** *Euploca*, *Heliotropium*, morphology, Thai flora, typification

## Abstract

A synopsis of Heliotropiaceae in Thailand is presented and, as part of this, the taxonomic history, identification keys, distribution maps, herbarium specimen citations and diagnostic characters are provided. Two genera and 12 species are recognised and four new combinations are proposed of *Tournefortia* under the genus *Heliotropium*, namely *H.hookeri*, *H.intonsum*, *H.montanum* and *H.ovatum*. Two species are newly recorded in Thailand: *Euplocaovalifolia* and *H.hookeri*. The neotype and lectotype are designated here for *Tournefortiamontana* and *T.boniana*, respectively.

## ﻿Introduction

Heliotropiaceae (= Boraginaceae s.l., subfamily Heliotropioideae) comprises ca. 450 species in four genera: *Heliotropium* L. (incl. *Tournefortia* L.), *Euploca* Nutt., *Myriopus* Small. and *Ixorhea* Fenzl (Diane et al. 2016; Luebert et al. 2016) and is widely distributed in the tropical, subtropical and temperate zones, with the centre of diversity in the Neotropics (Mabberley 2017). In the previous classification, based on morphology and anatomy, Förther (1998) recognised eight genera: *Argusia* Boehm., *Ceballosia* Kunkel, *Heliotropium*, *Hilgeria* Förther, *Ixorhea*, *Nogalia* Verdc., *Schleidenia* Endl. and *Tournefortia*. The distinct features of the family are based on scorpioid cymes and flowers with a terminal style, undivided, terminal with a conical stigmatic head having a basal ring-shaped stigma (Diane et al. 2016; Luebert et al. 2016). Molecular phylogenetic analyses of Heliotropiaceae, based on *trn*LUAA and ITS1 sequence data, have shown that *Heliotropium* and *Tournefortia* are not monophyletic (Diane et al. 2002; Hilger and Diane 2003). Based on a result of Hilger and Diane (2003), Tournefortiasect.Tournefortia is nested within *Heliotropium* s.s. which is a sister group to *Heliotropium* I clade and the group has a drupaceous fruit. The species of Heliotropiumsect.Orthostachys, *Hilgeria* and *Schleidenia* are within the *Euploca* clade. This relationship is also supported by morphological features, for example, the presence of bracts at the inflorescences and fruit breaking up into four distinct nutlets (Feuillet 2016). According to Hilger and Diane (2003), the molecular and morphological data suggest that a re-arrangement of the current classification is required. More new combinations of *Euploca* and *Heliotropium* have been made (e.g. Craven (2005); Melo and Semir (2009); Luebert et al. (2011); Feuillet (2016); Melo (2017); Frohlich et al. (2020); Antony and Javad (2020)).

In Southeast (SE) Asia, the contributions to the knowledge of *Tournefortia* and *Heliotropium* remain controversial. Many traditional *Tournefortia* species were recognised by Gagnepain and Courchet (1914), Ridley (1923), Johnston (1935), Backer and Bakhuizen van den Brink Jr (1965), Zhu et al. (1995) and Riedl (1997). Fletcher and Kerr (1951) reported the first checklist of the Heliotropiaceae species in the Florae Siamensis Enumeratio. The present study aims to: 1) provide a synoptic account of Heliotropiaceae for the Flora of Thailand, 2) present an identification key for species recognition and 3) propose new combinations of *Tournefortia* into *Heliotropium*.

## ﻿Materials and methods

Extensive collections of herbarium specimens at AAU, ABD, BK, BKF, BM, C, CMU, CMUB, E, K, K-W, KKU, L, QBG and SING (abbreviations follow Thiers 2022, continuously updated) were studied together with living material observations in the field. Species delimitation was based on the examination of original publications including the relevant literature: Clarke (1885); Gagnepain and Courchet (1914); Ridley (1923); Johnston (1935, 1951); Backer and Bakhuizen van den Brink Jr (1965); Zhu et al. (1995); Riedl (1997); Mill (1999). The type collections were observed online in JSTOR Global Plants (https://plants.jstor.org/). Most measurements were taken from dried specimens and spirit-preserved material and colour descriptions were studied from living material. Distribution, ecology and flowering and fruiting data were derived from specimen labels. All specimens were imported into SimpleMappr (Shorthouse 2010) to create distribution maps. All collections cited that have been seen by the authors are marked with ‘!’ and those seen as digital images are indicated with ‘image’.

The molecular data and morphological species concept (Hilger and Diane 2003; Diane et al. 2016; Luebert et al. 2016) were used in the present study. This concept is consistent with that of previous studies (i.e. de Candolle (1845); Gagnepain and Courchet (1914); Johnston (1935, 1951); Fletcher and Kerr (1951); Förther (1998)). Particular morphological features that were employed to delimit species in *Euploca* species are: stem position and branching, the presence of bracts in the inflorescence, cyme type and floral and fruit structure. For *Heliotropium*, features used include: habit, the presence of bracts in the inflorescence, cyme type and floral and fruit structures. For the generic circumscription, both morphological and molecular data are considered (Diane et al. 2002; Hilger and Diane 2003; Luebert et al. 2016). Morphologically, *Euploca* is separated from *Heliotropium* due to the bracteate inflorescence and fruit breaking up into four distinct nutlets. The terminology used in this present study is adapted from Weberling (1992) and Beentje (2012).

## ﻿Taxonomic treatment

### ﻿ Heliotropiaceae

Taxon classificationPlantaeBoraginalesHeliotropiaceae

Schrad., Commentat. Soc. Regiae Sci. Gött. Recent. 4: 192. 1819 nom. cons.

44ADB9EC-7FC9-5998-9285-B8375A94F313

#### Type genus.

*Heliotropium* L.

#### Description.

Annual or perennial herbs, climbing shrubs or small trees. Leaves simple, alternate, exstipulate, petiolate or sessile; lamina linear to ovate, apex acute to acuminate, margin entire or revolute. Inflorescences terminal or axillary, spike-like, scorpioid, subcapitate or subcorymbose cymes; with or without bracts. Flowers 5-merous, rarely 4-merous, bisexual, actinomorphic. Calyx 5-lobed, divided almost to the base, lobes linear to ovate, entire, persistent. Corolla white to pinkish-white, purple, pale green or greenish to yellowish, campanulate to funnel-shaped, lobes orbicular. Stamens 5, filaments adnate to the corolla tube. Pistil ovary superior, bicarpellate, usually 4-loculate, 1 ovule per locule, nectar disc at the base surrounds the ovary; style terminal, with a conical stigma structure forming a basal ring around style. Fruit dry or fleshy, separating into four 1-seeded nutlets or two 2-seeded nutlets. Seeds straight or curved, with endosperm.

Two genera and 12 species are found in Thailand.

### ﻿Key to the genera of Heliotropiaceae in Thailand

**Table:** 

1	Inflorescence bracteate; fruit separating into four nutlets	***Euploca***
—	Inflorescence ebracteate; fruit separating into two nutlets	***Heliotropium***

### ﻿ Euploca

Taxon classificationPlantaeBoraginalesHeliotropiaceae

Nutt., Trans. Amer. Philos. Soc., ser. 2, 5: 189. 1836.

96E76C6D-2C87-5FB0-BB39-3EB5C95A34CB

Heliotropium [unranked] Orthostachys R.Br., Prodr. Fl. Nov. Holland.: 493. 1810. Type species: Heliotropiumfoliatum R.Br.
Preslaea Mart., Nov. Gen. Sp. Pl. 2: 75. 1827, nom. illeg.
Heliotropium sect. Orthostachys (R.Br.) G.Don, Gen. Syst. 4: 361. 1838. Type species: Based on Heliotropium [unranked] Orthostachys R.Br.
Schleidenia Endl., Gen. Pl.: 646. 1839. Type species: Preslaeaparadoxa Mart.
Orthostachys (R.Br.) Spach, Hist. Nat. Vég. 9: 32. 1840. Type species: Based on Heliotropium [unranked] Orthostachys R.Br.
Hilgeria Förther, Sendtnera 5: 132. 1998. Type species: Hilgeriahypogaea (Urb. & Ekman) Förther.

#### Type species.

*Euplocaconvolvulacea* Nutt.

#### Description.

Annual herbs. Leaves petiolate or sessile; lamina linear to ovate, apex acute to obtuse, margin entire or revolute, pubescent to strigose or silky silver hairs on both surfaces. Inflorescences terminal or axillary, spike-like, scorpioid or subcapitate cymes, bracteate. Flowers 5-merous. Calyx 5-lobed, lobes linear to lanceolate. Corolla white with a yellow-orange throat inside, tubular or funnel-shaped, lobes orbicular to oblong, throat pubescent inside. Stamens included, sessile or with short filaments; anthers, elliptic-oblong. Ovary divided into 4 lobes. Fruits separating into four 1-seeded nutlets.

### ﻿Key to the species of *Euploca*

**Table:** 

1	Inflorescence spike-like or scorpioid cymes	**2**
—	Inflorescence subcapitate cymes	**4**
2	Leaves elliptic to obovate or oblanceolate; cymes 2-rowed scorpioid	**3. *E.ovalifolia***
—	Leaves linear to narrowly elliptic; cymes unilateral or spike-like	**3**
3	Prostrate much-branched stem; leaves narrowly elliptic with strigose hairs	**5. *E.strigosa***
—	Erect unbranched to few-branched stem; leaves linear with greyish tomentose hairs	**4. *E.paniculata***
4	Leaves elliptic with white pubescent hairs	**1. *E.bracteata***
—	Leaves narrowly lanceolate with stiff bristly white hairs	**2. *E.marifolia***

### ﻿1. Euploca bracteata

Taxon classificationPlantaeBoraginalesHeliotropiaceae

(R.Br.) M.W.Frohl. & M.W.Chase, Phytotaxa 434(1): 15. 2020.

740FC187-BFA0-567C-BBFD-66B9706BDD26

Fig. 2A

Heliotropium bracteatum R.Br., Prodr. Fl. Nov. Holland.: 493. 1810. Type: Australia, Northern Territory, Groote Eylandt, 15 Jan 1803, *Brown 2926* (lectotype, designated by Craven 1996, p. 628: BM [BM001040580!]; isolectotypes: K [K000998271!, K000998272!]).
Heliotropium marifolium var. bracteatum (R.Br.) M.R.Almeida, Fl. Maharashtra 3A: 287. 2001.
Heliotropium bracteatum var. leptostachyum Benth., Fl. Austral. 4: 451. 1869. Type: Australia, Cape York, Daemel s.n. (holotype K [K000998274!]; isotype BM [BM001040578!]).

#### Type.

Based on *Heliotropiumbracteatum* R.Br.

#### Distribution.

**Figure 1.**
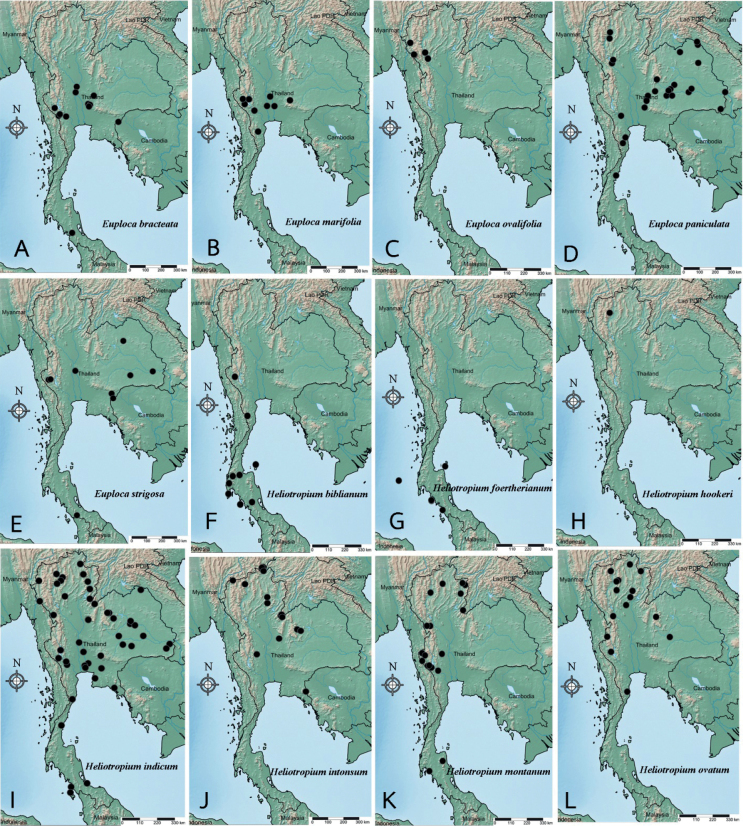
Distribution of Heliotropiaceae from Thailand, based on the specimens examined.

Pakistan, India, Sri Lanka, Myanmar, China (Hainan, Kwangtung), Laos, Cambodia, Vietnam, Thailand (Fig. 1A), Malaysia, Java, Australia.

#### Ecology.

Open areas in deciduous forest, dry secondary forest; 50–300 m alt., flowering and fruiting from February to October.

#### Specimens examined.

Thailand, Northern: Nakhon Sawan, 10 km north-west of Nakhon Sawan, 150 m alt., 21 July 1973, *Murata et al. T-16587* (BKF, L). South-western: Uthai Thani, Tapoh, 4 Jan 1962, *Larsen 9135* (C, K); Kanchanaburi, Sai Yok, 15 Aug 1967; Khao Pu Maklai, Nong Hoi, 10 July 1978, *Phengklai et al. 4247* (BKF, K); ibid., 40 m alt., 8 July 1922, *Marcan 889* (BM, K); Khao Tok, 50 m alt., 13 July 1930, *Kerr 19547* (BM, BK, K, E); ibid., 50 m alt., 10 July 1930, *Kerr s.n.* (K); Wangpho, 15 Oct 1967, *Chersmsirivathana 790* (BK). Central: Lop Buri, Lam Narai, 13 Nov 1975, *Smitinand 12108* (BKF); ibid., 22 Aug 1975, *Boonkurd 272* (BK); Saraburi, Phraphutthabat, 18 Sept 2004, *Pooma et al. 4798* (BKF); Phu Khae, 100 m alt., 26 June 1947, *Bunpheng 147* (BKF, K); Mae Nam Sak, 40 m alt., 29 May 1923, *Kerr 7020* (BK, K); South-eastern: Sa Kaeo, Aranyaprathet, 9 Apr 1930, *Kerr 19593* (ABD, BM, BK, BKF, E, K, L); ibid., 20 Oct 1828, *Put 2073* (BK, BM). Peninsular: Trang, Palian, 20 m alt., 26 May 1976, *Smitinand 12229* (BKF).

#### Diagnostic characters.

*Euplocabracteata* is suberect, with sparsely white pubescent hairs on both leaf surfaces, subcapitate cyme inflorescence at the end of branches and sessile flower or with pedicels up to 1 mm long. This species is similar to *E.marifolia*, sharing a similar habit, flowers and inflorescence form, but differs in having elliptic leaves (vs. linear to lanceolate leaves in *E.marifolia*).

### ﻿2. Euploca marifolia

Taxon classificationPlantaeBoraginalesHeliotropiaceae

(J.Köenig ex Retz.) Ancy & P.Javad, Nord. J. Bot. 38(11): 2. 2020.

A52B6207-7B54-5A57-93F3-EE1AC1A2DB98

Fig. 2B

Heliotropium marifolium J.Köenig ex Retz., Observ. Bot. 2: 8. 1781. Type: Asia, 1781, *Köenig 7052* (holotype LD [LD1748458 image!]).
Heliotropium scabrum Retz., Observ. Bot. 2: 8. 1781. Type: India, Coromandel coast, Köenig s.n. ([C10008736!]).
Euploca scabra (Retz.) M.W.Frohl. & M.W.Chase, Phytotaxa 434: 19. 2020. Type as above.

#### Type.

Based on *Heliotropiummarifolium* J.Köenig ex Retz.

#### Distribution.

Pakistan, India, Sri Lanka, China, Cambodia, Vietnam, Thailand (Fig. 1B), Peninsular Malaysia, Java.

#### Ecology.

In the open deciduous forest, dry secondary forest; 50–300 m alt., flowering and fruiting from January to October.

#### Specimens examined.

Thailand, Eastern: Nakhon Ratchasima, Nong Sarai, 300 m alt., 5 Sept 1963, *Smitinand & Sleumer 8359* (K, L). South-western: Kanchanaburi, Si Sawat, 16 Nov 1917, *van Beusekom et al. 3809* (BKF, L); Sai Yok, 15 Aug 1967; Hin Lat, 50 m alt., 28 Nov 1957, *Smitinand 3875* (BKF); Khao Ai Mao, 15 Nov 1968, *Sangkhachand 1576* (BKF, K); ibid., 40 m alt., 8 July 1922, *Marcan 889* (BM, K); Thong Pha Phum, 215 m alt., 6 Aug 2012, *Middleton et al. 5261* (BKF, E); Phetchaburi, Kaeng Krachan NP, 285 m alt., 11 Aug 2002, *Middleton et al. 972* (BKF, E, K). Central: Sing Buri, Mueang, 5 Jun 1880, *Put 2614* (BM, BK, K, BKF), Suphan Buri, Bang Plama, 22 Sept 1930, *Kerr s.n.* (K); Phra Nakhon Si Ayutthaya, 9 Sept 1922, *Marcan* 1000 (ABD, BM, K).

**Figure 2.**
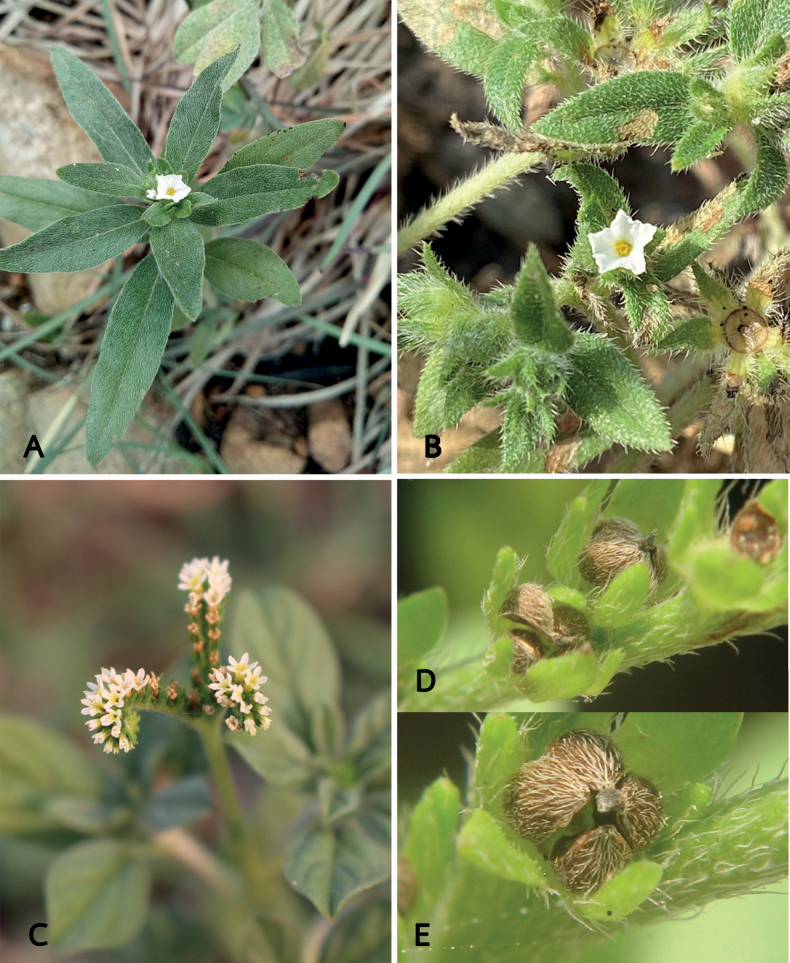
Species of *Euploca* from Thailand **A***E.bracteata* (R.Br.) M.W.Frohl. & M.W.Chase **B***E.marifolia* (J.Köenig ex Retz.) Ancy & P.Javad **C–E***E.ovalifolia* (Forssk.) Diane & Hilger **C** inflorescence **D** dehiscing fruit separating into four nutlets **A** Photo by Pramote Triboun **C–E** Photos by Siriporn Zungsontiporn.

#### Diagnostic characters.

*Euplocamarifolia* is characterised by being prostrate, much-branched with stiff bristly white hairs on the stems and leaves, lanceolate leaves (6–17 × 1–4 mm), revolute margin and inflorescence with leaf-like bracts.

### ﻿3. Euploca ovalifolia

Taxon classificationPlantaeBoraginalesHeliotropiaceae

(Forssk.) Diane & Hilger, Bot. Jahrb. Syst. 125(1): 48. 2003.

DA33899F-5A9B-5CFF-B5EE-E900BE173F30

Fig. 2C–E

Heliotropium ovalifolium Forssk., Fl. Aegypt.-Arab.: 38. 1775. Type: Yemen, Al-Hadiyah [Hadïe], *Forsskål, 299* (holotype C [C10002362!], isotype BM [BM000795522!]).
Heliotropium coromandelianum J.Köenig ex Retz., Observ. Bot. 2: 9. 1781. Type: India, Köenig s.n. (holotype C [C10008743!]; isotype BM [BM000795508!]).
Heliotropium gracile R.Br., Prodr. Fl. Nov. Holland.: 493. 1810.
Heliotropium ovalifolium ver. gracile (R.Br.) Domin, Biblioth. Bot. 22(89): 546. 1928. Type: Australia, Northern Territory, North Island, 19 Dec 1802, *Brown 2924* (lectotype, designated by Craven 1996, p. 559: BM [BM001040588!]; isolectotypes: K [K000998264!, K000998263!] GH [GH00097831]).

#### Type.

Based on *Heliotropiumovalifolium* Forssk.

#### Distribution.

Tropical Africa, Madagascar, Arabian Peninsula, Pakistan, India, Laos, Cambodia, Vietnam, Thailand (Fig. 1C), Australia, Solomon Islands.

#### Ecology.

Open area, sandy soil on riverbanks, rice fields, grassland, along roadsides; ca. 200 m alt., flowering and fruiting from December to May.

#### Specimens examined.

Thailand, Northern: Lamphun, Mae Ping NP, 22 May 2019, *Thammarong et al. 673* (QBG); Tak, Tha Song Yang-Mae Sa Riang, Moei river, 22 Mar 2006, *Pooma et al.* 6220 (BKF, L); Bhumibol Dam, 200 m alt., 29 May 2008, *Pooma et al.* 7073 (BKF); Ban Maesong, 23 June 2005, *Pooma et al.* 5443 (BKF); Mae Sa Riang-Mae Sot road, 15 May 2007, *Pooma et al. 6786* (BKF, E).

#### Vernacular.

Nguang chang dok khao (งวงช้างดอกขาว).

#### Diagnostic characters.

*Euplocaovalifolia* is newly recorded for Thailand. It is recognised by elliptic to obovate or oblanceolate leaves with silky silver hairs, spike-like or scorpioid cymes inflorescence with pedicels up to 2 cm long and a white corolla with a yellowish to yellow centre. This species is similar to *Heliotropiumindicum* in its inflorescence form, but differs in its leaf shape, bracteate inflorescences (vs. ebracteate in *H.indicum*) and fruit breaking up into four nutlets (vs. breaking up into two nutlets in *H.indicum*).

### ﻿4. Euploca paniculata

Taxon classificationPlantaeBoraginalesHeliotropiaceae

(R.Br.) M.W.Frohl. & M.W.Chase, Phytotaxa 434(1): 18. 2020.

BE176448-E55F-59B1-B557-7A24B348EE18

Fig. 3A–C

Heliotropium paniculatum R.Br., Prodr. Fl. Nov. Holland.: 494. 1810. Type: Australia, Queensland: Sweer`s lsland, 28 Nov 1802, *Brown 2930* (lectotype, designated by Craven 1996, p. 561: BM [BM001040573!]; isolectotype: K [K000998298!]).
Heliotropium tenunifolium var. paniculatum (R.Br.) Domin, Biblioth. Bot. 89: 549. 1928.
Heliotropium zeylanicum subsp. paniculatum (R.Br.) Kazmi, J. Arnold Arbor. 51: 156. 1970.

#### Type.

Based on *Heliotropiumpaniculatum* R.Br.

#### Distribution.

Pakistan, India, Myanmar, Thailand (Fig. 1D), Cambodia, Australia.

#### Ecology.

Open area of bare rock in dry dipterocarp and dry open deciduous forests, roadsides or wheat field; 50–1,000 m alt., flowering and fruiting from June to December.

**Figure 3.**
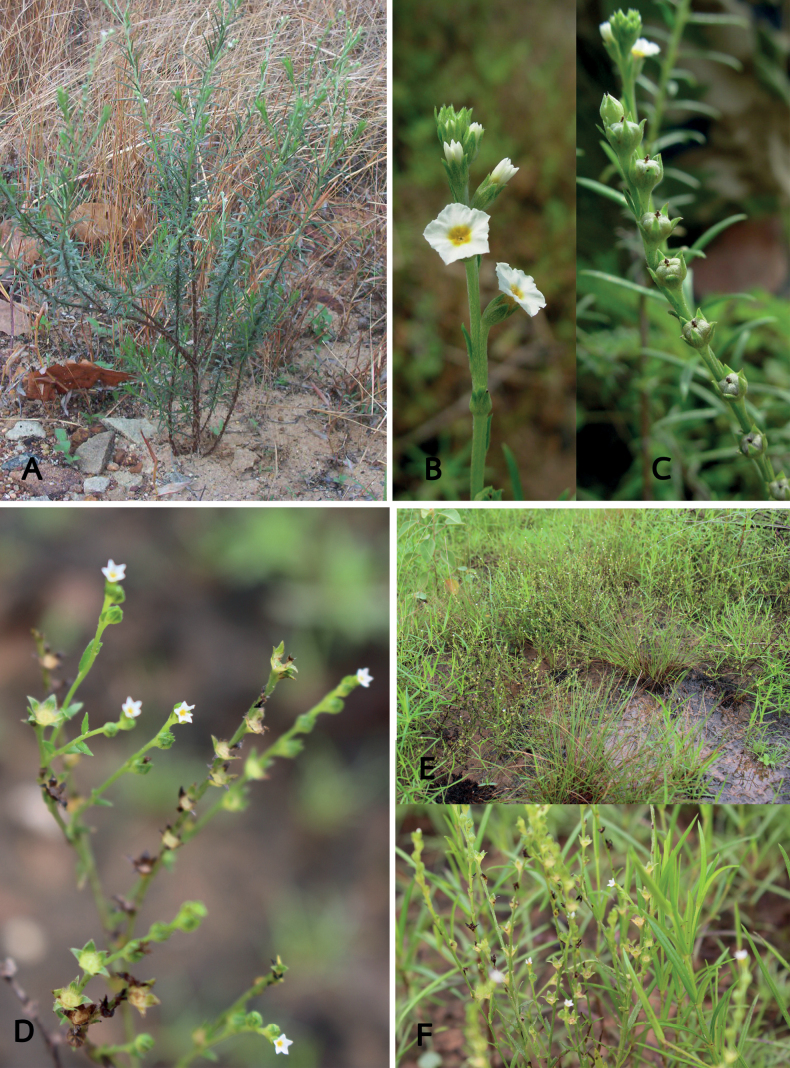
Species of *Euploca* from Thailand **A–C***E.paniculata* (R.Br.) M.W.Frohl. & M.W.Chase **A** habit and inflorescences **B** inflorescence **C** young fruits **D–F***E.strigosa* (Willd.) Diane & Hilger **D** inflorescence **E** habit **F** habit and inflorescences. Photos by Kanokorn Rueangsawang.

#### Specimens examined.

Thailand, Northern: Chiang Mai, 300 m alt., 19 Jan 1914, *Kerr 3291* (BM, K); Tak, Ban Tak, 150 m alt., 1 Sept 2016, *Pooma & Pattharahirantricin 7944* (BKF); Nakhon Sawan, 31 km north, 100 m alt., 2 June 1959, *Smitinand 5815* (BKF); Ban Dong Lan, Wang Jan, Sam Ngao, 28 Aug 2010, *Norsaengsri 7030* (QBG); Lamphun, Phra Puttabat Tak Pa, 24 Aug 2010, *Norsaengsri 7205* (QBG). North-eastern: Udon Thani, Ban Phen, 24 Dec 1964, *Chermsirivathana 225* (BK); Sakon Nakhon, Phu Phan NP, 400 m alt., 6 Aug 2004, *Nielsen et al. 1542* (BKF); Bueng Kan, Ban Tong, Seka, 220 m alt., 20 May 2004, *Pooma et al. 4116* (BKF); Tham Noi waterfall, 320 m alt., 30 July 2008, *Pooma et al. 7345* (BKF); Khon Kaen, Mueang Phon, 200 m alt., 5 July 1967, *Smitinand 12385* (BKF). Eastern: Chaiyaphum, Pa Khok Yai Chiu, 190 m alt., 28 Aug 1966, *S.N. 235* (BKF); Nong Bua Deng, 400 m alt., 15 Aug 1972, *Larsen et al. 31869* (AAU, BKF, E, K); Nakhon Ratchasima, Bannot Phattana, Road 226, km 42–41, 14 Sept 2004, *Pooma et al. 4677* (AAU, BKF, E); Phimai, 200 m alt., 26 Aug 1958, *Smitinand 4783* (BKF); ibid., 5 Aug 1968, *Pradit 316* (BK); Bua Yai, 200 m alt., 19 May 1931, *Kerr 20488* (ABD, BK, BKF, BM, K, L); Ban Chum Seng, 22 May 1929, *Noe 153* (BM, E, K); Surin, Chumphon Buri, 8 June 1982, *Paisooksantivatana & Sutheesorn y 913*–*82* (BK); Roi Et, Kaset Wisai, 150 m alt., 22 June 1969, *Simtinand & Nalamphun 10719* (BKF); ibid., 9 June 1982, *Paisooksantivatana & Sangkhachand y1000*–*82* (BK); Ubon Ratchathani, Khua Nangnee waterfall, Pha Taem NP, 200 m alt., 22 Aug 2001, *Pooma et al. 2281* (BKF), Phu Chong Nayoi NP, 20 Oct 2009, *Middleton et al. 5196* (BKF, E, K); ibid., 250 m alt., 20 May 1998, *Suksathan 1043* (QBG) ibid., 100 m alt., 25 Jan 1924, *Kerr 8345* (BK, BM, K). South-western: Kanchanaburi, Khao Meng, 14 Apr 1965, *Chantanamuck 1067* (BK); 50 m alt., 13 Sept 1931, *Kerr 20552* (BK, BM, E, K, L); ibid., 50 m alt., 13 Sept 1931, *Kerr 20553* (E, L); Prachinburi, Huai Saai, Cha-am, 19 Aug 2003, *Puudjaa 1222* (BKF); Wat Luke Chang, Luke Chang Village, Tha Mai Ruak Subdistrict, Tha Yang, 5 July 2007, *Maxwell 07*–*410* (QBG); Prachuap Khiri Khan, Hua Hin, 31 July 1976, *Maxwell 76*–*455* (AAU, BK, L); Khao Tao, Hua Hin, *Simitinand 1459* (BKF); ibid., 10 Nov 1928, *Marcan 2450* (BM, K); ibid., 20 m alt., 9 Nov 1928, *Kerr 16164* (AAU, BK, BM, K). Central: Lop Buri, Lam Narai, 50 m alt., 13 Nov 1975, *Smitinand 12102* (BKF); ibid., 50 m alt., 13 Nov 1975, *Smitinand 12109* (BKF); Phatthana Nikhom, Ban Diluang, km 29–30, 100 m alt., 17 May 2004, *Pooma et al. 3989* (BKF); Saraburi, 5 Sept 1954, *Bunnag 10* (BK). Peninsular: Chumphon, Bang Son, Pathio, 20 m alt., 10 Jan 1927, *Kerr 11351* (BK, BM, E, K, L).

#### Diagnostic characters.

*Euplocapaniculata* is erect with unbranched to few-branched stems that are woody at the base. The distinguishing features are linear leaves with greyish tomentose hairs, the spike-like inflorescences arranged in one rank that can elongate up to 20 cm and flowers with pedicels up to 2 mm long. The herbarium specimens from Thailand have mostly been confused with *E.strigosa*, but these two species can be differentiated by leaf shape and type of induments that are greyish tomentose (*E.paniculata*) and strigose (*E.strigosa*) on both leaf surfaces.

### ﻿5. Euploca strigosa

Taxon classificationPlantaeBoraginalesHeliotropiaceae

(Willd.) Diane & Hilger, Bot. Jahrb. Syst. 125(1): 49. 2003.

491C1319-2FAE-5A58-8DD6-8B6FF32E86D4

Fig. 3D–F

Heliotropium strigosum Willd., Sp. Pl., ed. 4, 1(2): 743. 1798. Type: Ghana [Guinea], Isert s.n. (holotype B [B-W 219/3253, microfiche], isotypes C [C10003972!], P [P-JU6571 image!]).
Lithospermum chinense Hook. & Arn., Bot. Beechey Voy.: 202. 1837. Type: China, Canton, *Vachell 286* (holotype E [E00369167!]; isotype BR [BR0000006966485 image!]).

#### Type.

Based on *Heliotropiumstrigosum* Willd.

#### Distribution.

Africa, Afghanistan, Pakistan, India, Nepal, Bhutan, Myanmar, Laos, Cambodia, Vietnam, China (Hainan, Kwangtung), Thailand (Fig. 1E), Malesia, Australia.

#### Ecology.

Open area of bare rock in dry dipterocarp and dry open deciduous forests; 50–400 m alt., flowering and fruiting from April to November.

#### Specimens examined.

Thailand, North-eastern: Sakon Nakhon, Phu Phan NP, 400 m alt., 6 Aug 2004, *Nielsen et al. 1542* (BKF); Phrae, Ban Nun, Song, 193 m alt., 25 June 2012, *Norsaengsri & Tathana 9480* (QBG). Eastern: Surin, Nadi, 17 May 1965, *Sakol 224* (BK); Ubon Ratchathani, Chiet, 100 m alt., 21 May 1932, *Kerr 21537* (BK, BM, E, K). Central: Chai Nat, Utapao, 20 Sept 1930, *Kerr 19692* (ABD, BM, BK). South-western: Kanchanaburi, 5 km west of Thong Pha Phum Town, 16 Oct 2015, *Tanming 873* (QBG); Thong Pha Phum, along route 323, 4 km, 240 m alt., 29 Nov 1982, *Koyama et al. T-30469* (BKF); Prachinburi, Watananakorn, 23 July 1987, *Paisooksantivatana & Sangkhachand 2109-87* (BK). South-eastern: Sa Kaeo, Aranyaprathet, 50 m alt., 3 Apr 1930, *Kerr 19575* (BM, BK, K). Peninsular: Songkhla, Padang Besar, 50 m alt., 24 Dec 1927, *Kerr 13602* (BM, BK, K).

#### Diagnostic characters.

*Euplocastrigosa* is most similar to *E.paniculata* in having spike-like inflorescences arranged in one rank that can elongate up to 10 cm long, but it differs in the stem being a prostrate to many-branched (vs. erect unbranched to few-branched stem in *E.paniculata*), the narrowly elliptic leaves with strigose hairs on both surfaces, 5–10 × ca. 3 mm (vs. linear leaves with greyish tomentose hairs, 7–40 × 1–2 mm in *E.paniculata*).

### ﻿ Heliotropium

Taxon classificationPlantaeBoraginalesHeliotropiaceae

L., Sp. Pl.: 130. 1753.

4DD2D184-6C7B-503F-B5C9-D92AFBBD3A4F

Tournefortia L., Sp. Pl.: 140. 1753. Type species. Tournefortiahirsutissima L. [= Heliotropiumverdcourtii Craven].
Argusia Boehm. in Ludwig, Def. Gen., ed. Boehmer: 507. 1760. Type species: Argusiasibirica (L.) Dandy.
Messerschmidia L. ex Hebenstr., Nov. Comm. Acad. Sci. Imp. Petrop. 8: 315, t. 11. 1763. Type species: Tournefortiasibirica L.
Tetrandra (DC.) Miq., Fl. Ind. Bat. 2: 928. 1858. Type species: Tetrandrawallichii Miq. [= Tournefortiatetrandra Blume].
Mallotonia (Griseb.) Britt., Ann. Missouri Bot. Gard. 2: 47. 1915. Type species: Mallotoniagnaphalodes (L.) Britton.

#### Type species.

*Heliotropiumeuropaeum* L.

#### Description.

Annual herbs, climbing shrubs or small trees. Leaves petiole; lamina ovate, elliptic to obovate or elliptic to lanceolate, apex acute to acuminate, obtuse or rounded, margin entire or irregularly undulate, strigose, stiff hairs or greyish-white tomentose on both surfaces. Inflorescences terminal or axillary, scorpioid cymes, subcorymbose or dichotomously branched, dense 2-rowed scorpioid cymes, ebracteate. Flowers 4–5-merous. Calyx 5-lobed, lobes lanceolate to ovate. Corolla pale green, white to pinkish-white, greenish to yellowish, with a yellow-orange throat inside, funnel-shaped or tube cylindrical, lobes broadly ovate to orbicular, throat glabrous inside. Stamens included, sessile or with short filaments; anthers ovate-lanceolate or elliptic-oblong. Ovary entire. Fruits drupe, deeply bilobed or distinctly lobed, mesocarp thinly fleshy or juicy, endocarp dividing into two 2-seeded nutlets.

### ﻿Key to the species of *Heliotropium*

**Table:** 

1	Annual herb or small tree; inflorescence spike-like, scorpioid, subcapitate or subcorymbose	**2**
—	Climbing shrub; inflorescence dichotomous branched, scorpioid cymes	**3**
2	Small trees; leaves 8–20 cm long with densely greyish tomentose hairs	**2. *H.foertherianum***
—	Annual herbs; leaves 3–6 cm long with strigose and stiff hairs	**4. *H.indicum***
3	Flower 4-merous; lower surface of leaves pale, brownish-black when dry	**1. *H.biblianum***
—	Flower 5-merous; concolorous leaves, light brown when dry	**4**
4	Leaves oblong-elliptic to ovate-lanceolate lanceolate, apex acute	**5**
—	Leaves ovate, apex long acuminate	**6**
5	Leaves oblong-elliptic; inflorescence dense dichotomous branched; corolla tube angular	***5. H.intonsum***
—	Leaves ovate-lanceolate; inflorescence loosely dichotomous branched; corolla tube not angular	**6. *H.montanum***
6	Leaves with minutely tubercules	**7. *H.ovatum***
—	Leaves with golden brown tomentose hairs	**3. *H.hookeri***

### ﻿1. Heliotropium biblianum

Taxon classificationPlantaeBoraginalesHeliotropiaceae

Craven, Blumea 50(2): 379. 2005.

797CD91A-4AAE-571D-9668-23B4F6C33ADF

Tournefortia tetrandra Blume, Bijdr. Fl. Ned. Ind. 14: 845. 1826. Type: Java, Blume s.n. (holotype L [L0281636!]).
Tournefortia wallichii DC., Prodr. 9: 527. 1845. Type: Singapore, Penang, *Wallich Numer. List 911* (holotype K-W [K00111261!]; isotypes K-W [K00111262!, K000998138!], M [M0188731 image!], GZU [GZU000106058 image!], MEL [MEL2502678 image!]).
Tournefortia tetrandra Blume var. angustifolia Moritzi, Syst. Verz. 52: 1845. Type: Java, *Zollinger 939* (lectotype, designated here: K [K000998147!]; isolectotypes: K [K000998148!], BM [BM001209066 image!], FI [FI009368 image!]).
Tetrandra zollingeri Miq., Fl. Ind. Bat. 2: 928. 1858. Type: Java, *Zollinger 395* (holotype K [K000998149!]).

#### Type.

Based on *Tournefortiatetrandra* Blume.

#### Distribution.

India (Nicobar Islands), Sri Lanka, Cambodia, Vietnam, Thailand (Fig. 1F), Malesia, New Guinea.

#### Ecology.

Deciduous forest, evergreen forest, 40–700 m alt., flowering and fruiting from April to December.

#### Specimens examined.

Thailand, South-eastern: Kanchanaburi, Thong Pha Phum, 700 m alt., 15 Dec 1995, *FRUD & van Welzen 111* (BKF). South-western: Prachuap Khiri Khan, Pa La U. trail, 260 m alt., 20 Jun 2004, *Middleton et al*. *2322* (BKF). Peninsular: Ranong, Kaper, 40 m alt., *Shimizu et al. T-26364* (AAU, BKF); ibid., 500 m alt., 19 June 1932, *Kerr 21710* (BK, BM, E, K); Surat Thani, Ko Pha-ngan, 10 Nov 1927, *Put 1247* (BM, BK, E, K); ibid., 50 m alt., 3 Mar 1927, *Kerr 12456* (BK, BM, E, L, K); Bandon, 2 Jan 1935, *Seidenfaden 2089* (K); Phangnga, 500 m alt., 9 Dec 1928, *S.N. 3994* (K, SING); Trang, Khao Pap Pa, 13 Mar 1974, *Larsen & Larsen 33277* (AAU); Yan Ta Khao, Sai Roong waterfall, 400 m alt., 26 Apr 1987, *Maxwell 87*–*434* (BKF, CMU, L); Phuket, Khao Pha Tao Non-Hunting Area, Bang Pae waterfall, 50 m alt., 14 Oct 2004, *Gardner & Sidisunthorn 44268* (QBG).

#### Vernacular.

Fa Ta Hueng (ฟ้าตาหึง).

#### Diagnostic characters.

*Heliotropiumbiblianum* can be recognised mainly by its tetramerous flower, acute apex leaf, thick leaves with minute tubercules on both leaf surfaces, corolla up to 8–10 mm long and globose fruit up to 8 mm in diameter. According to the label of Tournefortiatetrandravar.angustifolia, four duplicate specimens were originally collected and are preserved at BM, K and FI. The specimen at K [K000998147] is designated here as the lectotype because it is in the best condition.

### ﻿2. Heliotropium foertherianum

Taxon classificationPlantaeBoraginalesHeliotropiaceae

Diane & Hilger, Bot. Jahrb. Syst. 125(1): 46. 2003.

648634B1-319F-5E31-A8F5-7D3E9BF7F9D3

Fig. 4A–C

Tournefortia argentea L.f., Suppl. Pl.: 133. 1781. Type: Sri Lanka, Köenig s.n. (holotype BM [BM001014452 image!]).
Messerschmidia argentea (L.f.) I.M.Johnst., J. Arnold Arbor. 16: 164. 1935.
Argusia argentea (L.f.) Heine, Fl. Nouv.-Calédonie & Dépend. 7: 109. 1976.

#### Type.

Based on *Tournefortiaargentea* L.f.

#### Distribution.

China (Hainan), Taiwan, Japan (Ryukyu), Vietnam, Thailand (Fig. 1G), Malesia, New Guinea, Australia.

#### Ecology.

Sandy beaches, flowering and fruiting from November to February.

#### Specimens examined.

Thailand, Peninsular: Surat Thani, Ban Bua Put, Ko Samui, 14 May 1928, *Kerr 15728* (BK, E, K, TCD); Phangnga, Similan NP, 14 Nov 1996, *Santisuk s.n*. (BKF); Krabi, Lan Ta NP, 7 Jan 1992, *Niyomdham 2854* (BKF); Ko Lan Ta Yai, 20 Mar 1998, *Chamchumroon 37* (BKF); Satun, Adang, La-ngu, 15 Jan 1928, *Kerr 14095* (BK, K).

**Figure 4.**
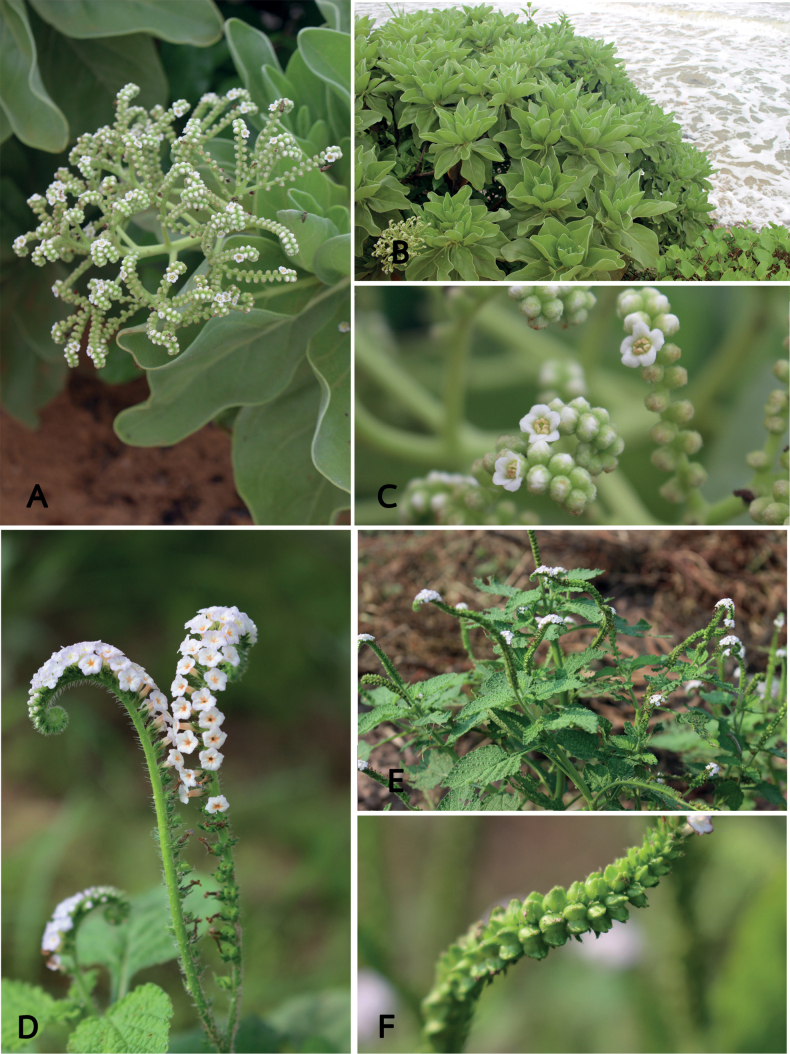
Species of *Heliotropium* from Thailand **A–C***H.foertherianum* Diane & Hilger **A** inflorescence **B** habit **C** flowers **D–F***H.indicum* L. **D** inflorescences **E** habit and inflorescence **F** young fruits. Photos by Kanokorn Rueangsawang.

#### Vernacular.

Nguang chang thale (งวงช้างทะเล).

#### Diagnostic characters.

*Heliotropiumfoertherianum* is recognised by the combination of a small tree, oblanceolate or obovate leaves that are up to 20 cm long and have densely greyish tomentose hairs, subcorymbose inflorescence with peduncles up to 8 cm long, corollas as long as the calyx and fleshy fruits with a fleshy mesocarp and spongy endocarp.

### ﻿3. Heliotropium hookeri

Taxon classificationPlantaeBoraginalesHeliotropiaceae

(C.B.Clarke) Rueangs. & Chantar. comb. nov.

E8A2F60A-7EFD-5C14-ADAB-4351317D4231

urn:lsid:ipni.org:names:77327199-1

Tournefortia hookeri C.B.Clarke, Fl. Brit. India 4: 147. 1883.
Tournefortia viridiflora Gamble, Darjeeling List: 57. 1878. nom.illeg.

#### Type.

Sikkim, Himalayas, *Hooker s.n*. (holotype K [K000998133!]).

#### Distribution.

East Himalaya, Myanmar, Thailand (Fig. 1H).

#### Ecology.

Deciduous forest, flowering and fruiting in June.

#### Specimens examined.

Thailand, Northern: Chiang Mai, Ban Pa Kar, Samoengtai, Samoeng, 1,424 m alt., 26 June 2008, *Jatupol 08*–*275* (QBG).

#### Diagnostic characters.

*Heliotropiumhookeri* is newly recorded for Thailand due to a specimen of this distinctive species seen from QBG. This species is recognisable in the leaves being broadly ovate with dense golden brown tomentose hairs on the lower surface and loosely dichotomous cymose.

### ﻿4. Heliotropium indicum

Taxon classificationPlantaeBoraginalesHeliotropiaceae

L., Sp. Pl.: 130. 1753.

CDD8B793-AD03-523E-8BD0-C88A3C27D5FE

Fig. 4D–F

#### Type.

India utraque, Herb. Hermann 1: 9, no.70 (lectotype, designated by Mill in Cafferty and Jarvis 2004, p. 802: BM [BM00061256!]).

#### Distribution.

North America, South America, Pakistan, India, Sri Lanka, China, Myanmar, Taiwan, Japan, Laos, Cambodia, Vietnam, Thailand (Fig. 1I), Sumatra, Malaysia, Java, Borneo, Australia.

#### Ecology.

Common on sandy soil near stream or open areas, rice fields, roadsides; 50–1,000 m alt., flowering and fruiting from January to April.

#### Specimens examined.

Thailand, Northern: Mae Hong Son, Mueang, 256 m alt., 10 Sept 2013, *Lakoet 0352* (QBG); 350 m alt., 18 June 1973, *Geesink et al. 5937* (AAU, BKF, E, K, TCD); Chiang Mai, Doi Sa Ket, 12 Dec 2007, *Warintorn 07*–*105* (QBG); San Kamphang, 325 m alt., 10 July 1996, *Panatkool et al. 37* (BKF); Doi Suthep, 300 m alt., *Hosseus s.n.* (K); ibid., 1,000 m alt., 12 May 1915, *Winit 402* (K, E); ibid., 1,000 m alt., 5 Jan 1911, *Kerr 1845* (BM, K, E); Hod, 8 Sept. 1996, *BGO Staff 28* (QBG); Ban Chang Keong, 488 m alt., 28 May 2019, *Pingyot 276* (QBG); Chiang Rai, Thoeng, 12 Feb 2011, *Norsaengsri & Tathana 7444* (QBG); San Pa Tong, 325 m alt., 26 Apr 1988, *Maxwell 88*–*521* (BKF, CMUB); Phayao, Ban Khun Kam Lang, Pong, 400 m alt., 19 May 2017, *Pongamorkul 6201* (QBG); Nan, Na Noi, 9 Apr 2018, *Khattiyot et al. 778* (QBG); Huai Haeng, 318 m alt., 26 Apr 2017, *Muaengyen 2011* (QBG); Lampang, Sop Prap, 28 Feb 2012, *Norsaengsri et al. 9055* (QBG); Uttaradit, Nam Pat, 22 Mar 2011, *Romklao Botanical Garden 0224/2554* (QBG); Tak, Mae Sot, 22 July 1959, *Floto 7653* (C); Tha Song Yang, 15 May 2007, *Pooma et al. 6785* (BKF, E, L); Phitsanulok, Tham Pha Phon Non-Hunting Area, 5 Mar. 2012, *Maknoi 4627* (QBG); Wat Hua Khao, 31 Jan 2012, *Maknoi 4565* (QBG). North-eastern: Loei, Na Haeo, Yaa Ngoung Chaang, 31 July 1995, *Nanakorn et al. 4077* (QBG); Phu Kradueng, 27 Aug 1988, *Tamura T-60450* (BKF); Sithan, Huai Yang, 300 m alt., 21 Feb 1958, *Ploenchit 1342* (BKF); Bueang Kan, Mueang, 27 Dec 2011, *Norsaengsri & Tathana 8581* (QBG); Maha Sarakham, Kosum Phisai, 3 May 2001, *Norsaengsri et al. 1440* (QBG); Ban Maong Ai, 130 m alt., 3 Apr 2001, *Norsangsri et al. 1440* (QBG); Chiang Yuen, 14 Apr 2018; Khon Kaen, Chum Phae, 2 Sept 1967, *Shimizu et al. T-8685* (BKF, E, TCD). Eastern: Nakhon Ratchasima, Bua Yai, 18 Mar 2009, *Norsaengsri 5135* (QBG); Ban Chum Sang, 25 May 1929, *Nai Noe 277* (ABD, BM, BK, K); Ubon Ratchatani, Khong Chiam, 170 m alt., 25 May 2001, *Greijmans 90* (BKF); Phibun Mangsahan, 250 m alt., 9 Dec 1982, *Koyama et al. T-30698* (AAU, BKF); Det Udom, 270 m alt., 10 Dec 1982, *Koyama et al. T-30778* (BKF); Buri Ram, 7 Aug 1970, *Sutheesorn 2065* (BK); Ban Na, Suwan Naphum, 16 Aug 1982, *Sutheesorn & Saraphunphichit 5354* (BK). South-western: Kanchanaburi, Si Sawat, 150 m alt., 6 Nov 1971, *van Beusekom et al. 3955* (BKF, C, K); Thum Pha, 13 Dec 1961, *Phengklai 228* (BKF); Kwae Noi, 30 Apr 1946, *Wichian 304* (K); Na Ta Hom, 23 Apr 1965, *Chantanamuck 1094* (BK); Prachuap Khiri Khan, 18 Aug 1967, *Shimizu et al. T-7689* (AAU, BKF, E, K). Central: Chat Nat, 5 Mar 1958, *Sørensen et al. 1922* (E, C); Pathum Thani, 50 m alt., 12 Mar 1958, *Smitinand 4444* (BKF); Nakhon Nayok, Khao Yai, 1,000 m alt., 5 May 1964, *R.S. 116* (BKF); 40 m alt., 6 Aug 1970, *Phengklai et al. 3748* (BKF); Nonthaburi, Sai Maa Tai, 14 Apr 1992, *Puudjaa 65* (BKF); Bangkok, 5 July 1920, *Marcan 293* (BM, K); 27 Oct 1919, *Kerr 3840* (ABD, BM, C, K); 19 Apr 1926, *Lakshnakara 52* (BK); Sept 1921, *Smith 49* (BK); 1899, *Zimmermann s.n.* (K); Ang Thong, 30 Dec 1929, *Put 2595* (AAU, ABD, BK, BM, K). South-eastern: Chachoengsao, Phanom Sarakham, 50–100 m alt., 1 Oct 1984, *Murata et al. T-37042* (BKF); Chon Buri, Si Racha, 30 Nov 1927, *Collins 2025* (ABD, BM, BK, K, TCD); Chanthaburi, 8 Apr 1959, *Sørensen et al. 7214* (C). Peninsular: Chumphon, 31 Jan 1958, *Sørensen et al. 829* (BKF, C); Ranong, Kapoe, 19 Jan 2016, *Kertsawang 3937* (QBG); Satun, La-ngu, Ko Kabeng, 11 Apr 2003, *Phengklai et al. 13650* (BKF); Songkhla, 21 Aug 1995, *Larsen et al. 45836* (AAU, BKF); Satun, Terutao, 5 Mar 1966, *Hansen & Smitinand 12542* (BKF, C).

#### Vernacular.

Ya nguang chang (หญ้างวงช้าง).

#### Diagnostic characters.

*Heliotropiumindicum* is likely native to tropical America and introduced in all the tropical regions of the world. This species is also a common weed with medicinal properties (Dash and Abdullah 2013). It is most easily recognised by ovate to elliptic leaves with strigose and stiff hairs on both surfaces, truncate or obtuse leaf base, irregularly undulate leaf margins, white to purple or purplish corollas with a yellow-orange throat inside and fruits are deeply 2-lobed with apical divergent lobes.

### ﻿5. Heliotropium intonsum

Taxon classificationPlantaeBoraginalesHeliotropiaceae

(Kerr) Rueangs. & Chantar. comb. nov.

053C2173-7696-52D8-A810-92CB54313975

urn:lsid:ipni.org:names:77327200-1

Fig. 5A

Tournefortia intonsa Kerr, Bull. Misc. Inform. Kew 1940: 185. 1940.

#### Type.

Thailand, Doi Sutep, ca. 900 m alt., evergreen forest, *Kerr 2285* (holotype K [K000998142!]; isotypes BM [BM001209041!], E [E00766166!]).

#### Distribution.

Laos, Thailand (Fig. 1J).

#### Ecology.

In deciduous forest, evergreen forest, flowering and fruiting from November to February.

#### Specimens examined.

Thailand, Northern: Mae Hong Son, Pang Mapha, 580 m alt., 26 Feb 1968, *Hansen & Smitinand 12741* (AAU, BKF, C, E, K); Chiang Mai, Doi Chiang Dao, 20 Mar 1956, *Suvarnakoses 1121* (BKF, K); ibid., 700 m alt., 18 Feb 1958, *Sørensen et al. 1312* (BKF); ibid., 23 Dec 1931, *Put 4570* (BK, E, K); ibid., 600–800 m alt., 14 Mar 1956, *Garrett 1449* (K, L); ibid., 600 m alt., 14 Mar 1956, *Garrett 1476* (K, L); ibid., 1,500 m alt., 17 Feb 1958, *Smitinand 4218* (BKF); ibid., 525 m alt., 26 Feb 1989, *Maxwell 89*–*273* (BKF, CMU, CMUB, L); ibid., 550 m alt., 10 Mar 1990, *Maxwell 90*–*294* (CMU); Chiang Rai, Doi Tung, 750 m alt., 15 Feb 2012, *van de Bult 1241* (BKF); Mae Sai, Huai Nam Dang, 612 m alt., 13 Jan 2011, *Norsaengsri & Tathana 7537* (BKF, QBG); Mae Sai, Ban Phamee, 572 m alt., 15 Feb 2012, *Norsaengsri & Tathana 9003* (QBG); Hua Yane, Mae Chan, 25 Jan 1970, *Sutheesorn 1438* (BK); Doi Tung, 1,400 m alt., 29 Jan 1989, *Bragg 8* (CMU, L); Phrae, Mae Khaen stream, 440 m alt., 5 Jan 1972, *van Beusekom, et al. 4636* (C, BKF, K); ibid., 22 Mar 1912, *Vanpruck 454* (K); Mae Yom NP, 400 m alt., 15 Dec 1993, *Maxwell 93*–*1509* (CMUB, L)]. North-eastern: Phetchabun, Chon Daen, 15 Jan 1969, *Vacharapong 304* (BK); Loei, Phu Suan Sai NP, 11 Mar 2008, *Maknoi 2029* (BKF, QBG); route to Sam Nuck Bab, 15 May 2008, *Maknoi & Srisaga 2302* (BKF, QBG); Tad Hueang waterfall, 1,000 m alt., 16 May 2006, *Maknoi 824* (QBG); Na Haeo, 650 m alt., 27 Apr 1995, *Nanakorn et al. 3239* (QBG); Khon Kaen, Pha Bhroung Cave, Chum Phae, 14 Feb 1963, *Chantanamuck 295* (BK). South-western: Uthai Thani, Huai Haeng, 27 Apr 1963, *Kasem 329* (BK). South-eastern: Chanthaburi, Pong Nam Ron, 600 m alt., 12 Jan 1956, *Smitinand 3192* (BKF).

**Figure 5.**
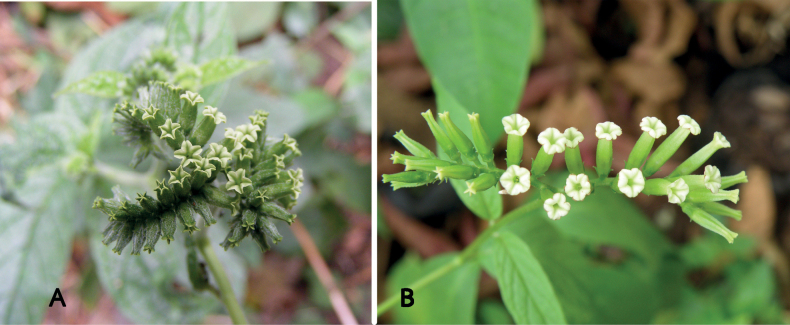
Species of *Heliotropium* from Thailand **A***H.intonsum* (Kerr) Rueangs. & Chantar. **B***H.montanum* (Lour.) Rueangs. & Chantar. Photos by Kanokorn Rueangsawang.

#### Vernacular.

Ya Nguang Chang Luang (หญ้างวงช้างหลวง).

#### Diagnostic characters.

*Heliotropiumintonsum* is most similar to *H.montanum* in its dichotomous branched inflorescences and subsessile pedicels, but differs in the leaves being oblong or elliptic with densely strigose hairs (vs. glabrous to glabrescent ovate-lanceolate leaves in *H.montanum*) and both the inflorescences and corollas bear densely greyish tomentose hairs (vs. glabrous in *H.montanum*).

### ﻿6. Heliotropium montanum

Taxon classificationPlantaeBoraginalesHeliotropiaceae

(Lour.) Rueangs. & Chantar. comb. nov.

DFB93912-0AF1-59E8-ACD2-3C792D85A2D6

urn:lsid:ipni.org:names:77327201-1

Fig. 5B

Tournefortia montana Lour., Fl. Cochinch. 1: 122. 1790.
Messerschmidia montana (Lour.) Roem. & Schult. Syst. Veg., ed. 15 bis 4: 544. 1819.
Tournefortia sampsonii Hance, J. Bot. 6: 330. 1868. Type: China, Sai-chii-shan, Cantonesis, *Sampson 13035* (holotype BM [BM001014454!]).
Tournefortia boniana Gagnep., Notul. Syst. (Paris) 3: 33. 1914. Type: Vietnam, Tonkin mèridional, *Bon 1932*, (lectotype, designated here: P [P03897617 image!]; isolectotypes: P [P03897616 image!], K [K000998141!]).
Tournefortia gaudichaudii Gagnep., Notul. Syst. (Paris) 3: 34. 1914. Type: Vietnam, Cochinchine: Tourane, *Gaudichaud 180* (lectotype, designated here: P [P03897604 image!]; isolectotypes: P [P03897602 image!], K [K000998140!]).
Tournefortia brachyantha Merr. & Chun, Sunyatsenia 2: 314. 1935. Type: China, Jiangsu, Hainan, *How70424* (holotype K [K000998121!]).

#### Type.

Vietnam, Cochinchine: Tourane, *Gaudichaud 180* (neotype, designated here: P [P03897604 image!]; isoneotypes: P [P03897602 image!], K [K000998140!]).

#### Distribution.

India (Assam), Bangladesh, southern China, Myanmar, Laos, Vietnam, Cambodia, Thailand (Fig. 1K).

#### Ecology.

Open roadside, along the streams in evergreen forest, bamboo forest, slopes of the hills in evergreen forest and mixed deciduous forests, 250–1,500 m alt., flowering and fruiting from February to December.

#### Specimens examined.

Thailand, Northern: Chiang Rai, Mae Chan, 450 m alt., 17 Mar 2005, *Pooma et al. 4859* (BKF); Chiang Mai, Mae Hia Thai Literary Botanical Garden, 350 m alt., 13 June 2003, *TLBG 165* (QBG); 300 m alt., 21 Jan 1921, *Kerr 4692* (BM, BK, K); Nan [Doi Phu Kha NP 1,500 m alt., 24 Jan 2003, *Srisanga 2684* (QBG); ibid., 11 Apr 2002, *Srisanga 1253* (QBG); ibid., 1,200 m alt., 19 Mar 2000, *Srisanga 1340* (BKF, QBG); Ban Den Thara, Pra That, Chiang Klang District, 346 m alt., 15 Mar 2018, *Khattiyot et al. 616* (QBG); Tam Paa Toop, Ta Wang Paa, 10 km from Nan, 360 m alt., 24 Feb 1998, *Srisanga et al. 220* (QBG); Lampang, Doi Luang NP, Wahng Gaye waterfall, 625 m alt., 26 Mar 1997, *Maxwell 97-753* (BKF); Uttaradit, Phu Soi Dao NP, 1,000 m alt., 18 Mar 2002, *Chamchumroon & Puff V.C.1438* (BKF); Tak, Doi Muser, Hui Sakulee, 700 m alt. 27 Feb 1987, *Paisooksantivatana Y2032-87* (BK); Mae Sot to Mae Sariang, 19 Jan 1995, *Pooma 1007* (BKF, CMUB). South-western: Uthai Thani, Ban Rai, 4 Mar 1977, *Sutheesorn 4017* (BK); Kanchanaburi, Sangkhla Buri to Thong Pha Phum, 180 m alt., 17 Dec 2009, *Pooma et al. 7436* (BKF); Sai Yok, Khwae Noi, 21 Dec 1961, *Larsen 8893* (BKF, C, K); ibid., 21 Dec 1961, *Simitnand 9500* (BKF); Sai Yok, 23 Dec 1961, *Larsen 8916* (C); Pompee Village near Khwae Noi, 250 m alt., 26 Mar 1968, *van Beusekom & Phengklai 129* (BKF, E, K, L); Mae Nam Noi near waterfall, 2 Jan 1962, *Phengklai 398* (BKF); Huai Banlcae, 8 Nov 1971, *van Beusekom et al. 3528* (BKF); Thong Pha Phum NP, 260 m alt., 3 Dec 2003, *Sirimongkol 69-2* (BKF). Peninsular: Krabi, Ban Nai Chong, 17 Dec 1965, *Umpai 208* (BK); Khap Thong Thai, 19 Jan 1966, *Hansen & Smitinand 12002* (BKF, C); Nakhon Si Thammarat, Nop Phitum, 80 m alt., 13 Feb 2005, *Williams et al. 1475* (BKF, E).

#### Diagnostic characters.

*Heliotropiummontanum* can be recognised by ovate-lanceolate leaves, glabrous to glabrescent on both surfaces and minute tubercules on the lower surface, loosely dichotomous branched inflorescences and glabrous flowers. The specimens from Thailand have mostly been misidentified as *H.intonsum*. These two species can be differentiated by leaf shape, the type of indument on each of the inflorescences and corollas that are glabrous (*H.montanum*) or densely greyish tomentose hairs (*H.intonsum*).

In the original description of *T.montana*Loureiro (1790) described from ‘*Cochinchina tributarii*’, the type collection of Loureior has not been found in either P or BM. Therefore, this species is represented by specimens of *Gaudichaud 180* (K000998140, P03897604, P03897602) from ‘*Cochinchine*: *Tourane*’, Loureiro’s initial locality. The specimen at P [P03897604] is designated here as a neotype for *T.montana* because it is in the best condition and the characters match the original description.

In the protologue, Gagnepain (1914) described *T.boniana*, based on two different collections (*Bon 1357 & Bon 1932*). *Bon 1932* P [P03897617] is chosen as the lectotype because it is best preserved, with complete inflorescences and numerous flowers and has two duplicates.

Gagnepain (1914) did not specify which of the duplicates of *T.gaudichaudii* was the holotype; therefore, the specimen at P [P03897604] is designated as the lectotype because it is a perfect match for the description in the protologue.

### ﻿7. Heliotropium ovatum

Taxon classificationPlantaeBoraginalesHeliotropiaceae

(Wall. ex G.Don) Rueangs. & Chantar. comb. nov.

7AFF3525-C2FD-53FF-8F67-689AD23D37A5

urn:lsid:ipni.org:names:77327202-1

Tournefortia ovata Wall. ex G.Don, Gen. Hist. 4: 369. 1837.

#### Type.

Myanmar, Rangoon, *Wallich Numer*. *List 908* (holotype K [K001110253!]).

#### Distribution.

India (Andaman Islands, Nicobar Islands), Myanmar, Thailand (Fig. 1L).

#### Ecology.

Open or shaded, slightly disturbed area in mixed evergreen forest 400–500 m alt., flowering and fruiting from November to April.

#### Specimens examined.

Thailand, Northern: Chiang Rai, Tham Luang-Khun Nam Nang Norn NP, Mae Sai, 560 m alt., 27 Mar 2012, *Norsaengsri & Tathana 9227* (QBG); Mae Tak, 3 Mar 1958, *SØrensen et al. 1865* (BKF); Chiang Mai, Gnai, Tintok, 500 m alt., 8 Mar 1965, *C.A. & B.S. 270* (BKF); Doi Chiang Dao, 750 m alt., 29 Mar 1995, *Maxwell 95*–*269* (BKF); ibid., 575 m alt., 11 Mar 1989, *Maxwell 89*–*317* (BKF, CMU, L); ibid., 550 m alt., 8 Mar 1965, *Hambanond 270* (BK); Phayao, Doi Pha Dam, Rom Yen Subdistrict, Chiang Kham, 890 m alt., 21 Feb 2013, *La-ongsri et al., 2662* (QBG); Lampang, Mae Pukaung, 6 Mar 1925, *Winit 1282* (BK, K); Nan, Song Khwae, Nam Pan Village, 350 m alt., 12 Jan 2011, *Srithi 630* (QBG); Tak, Lan Sang NP, 2 Jan 1969, *Cheviwat & Nimanong 10* (BKF); Phrae, 500 m alt., 22 Mar 1913, *Vanpruck 454* (BKF, K); Huai Rai, 13 Mar 1961, *Chanthamuk 6* (BKF); ibid., 13 Mar 1961, *Chanthamuk 9* (BKF); ibid., 23 Mar 1961, *Phengklai 66* (BKF, C, K); 26 Jan 1913, *Vanpruck 357* (BKF, K); Sukhothai, Srisatchanalai NP, route to Tad Dao Waterfall, 3 Feb 2015, *Maknoi 7243* (QBG). North-eastern: Phetchabun, Nam Nao NP, 18 Feb 2014, *Maknoi 6553* (QBG). Eastern: Nakhon Ratchasima, Bua Yai, 400 m alt., 29 Nov 1924, *Kerr 9487* (BM, BK, E, K, L). South-western: Kanchanaburi, Si Sawat, Dongyai, 50 m alt., 17 Aug 1971, *Phengklai et al. 3016* (BKF), ibid., 600 m alt., 17 Jan 1926, *Kerr 10233* (BM, BK, K, E); Thung Yai Naresuan WS, Sangklaburi, 475 m alt., 11 Apr 1994, *Maxwell 94*–*489* (CMUB, L); Prachuap Khiri Khan, Pran Buri, 25 Nov 1929, *Put 2449* (BM, BK, K).

#### Vernacular.

Liang (เหลียง).

#### Diagnostic characters.

*Heliotropiumovatum* is similar to *H.biblianum* in having ovate or ovate-lanceolate leaves, but differs in the 5-merous flower and densely minute tubercules on the lower leaf when dry (vs. 4-merous flowers and minute tubercules on both leaf surfaces in *H.biblianum*).

## Supplementary Material

XML Treatment for Heliotropiaceae

XML Treatment for Euploca

XML Treatment for Euploca bracteata

XML Treatment for Euploca marifolia

XML Treatment for Euploca ovalifolia

XML Treatment for Euploca paniculata

XML Treatment for Euploca strigosa

XML Treatment for Heliotropium

XML Treatment for Heliotropium biblianum

XML Treatment for Heliotropium foertherianum

XML Treatment for Heliotropium hookeri

XML Treatment for Heliotropium indicum

XML Treatment for Heliotropium intonsum

XML Treatment for Heliotropium montanum

XML Treatment for Heliotropium ovatum

## ﻿Additional information

### Author ORCIDs

Kanokorn Rueangsawang https://orcid.org/0000-0003-1595-1514

Pranom Chantaranothai https://orcid.org/0000-0002-5065-6169

### Data availability

All of the data that support the findings of this study are available in the main text.
